# Back to the future: Linking early psychiatric symptoms to transdiagnostic cognitive functioning in at-risk youth from the adolescent brain cognitive development study

**DOI:** 10.1016/j.jpsychires.2025.12.017

**Published:** 2025-12-09

**Authors:** C.J. Wang, D. Raucher-Chéné, K.M. Lavigne

**Affiliations:** a Department of Psychology, McGill University, Montreal, Quebec, Canada; b Douglas Research Centre, Montreal, Quebec, Canada; c Department of Psychiatry, McGill University, Montreal, Quebec, Canada

**Keywords:** High-risk, First-degree relatives, Hierarchical taxonomy of psychopathology, Children, Episodic memory, Reading

## Abstract

Cognitive impairment (problems in thinking, learning, remembering, judging, and decision-making) is central to many psychiatric disorders and may often appear well before the symptom onset. Given the moderate heritability of psychiatric disorders, children with first-degree relatives affected by severe mental illness are at higher risk and may show early psychiatric symptoms. Our study explored the potential association between cognitive functioning and early subsyndromal transdiagnostic psychiatric symptoms in at-risk youth. We compared 924 at-risk youth (aged nine to ten) with 924 matched controls from the Adolescent Brain Cognitive Development (ABCD) study. At-risk youth performed worse than controls in episodic memory, executive function, and working memory and exhibited more psychiatric symptoms (i.e., emotional dysfunction, psychosis and externalizing symptoms) than controls. Multivariate partial least squares in at-risk youth revealed a pattern linking heightened psychiatric symptoms with reduced cognitive performance across all domains except executive function, driven primarily by memory and language abilities, suggesting the well-established link between cognitive dysfunction and psychiatric symptoms is already present in at-risk youth, even prior to manifestation of clinically meaningful levels of cognitive impairment or psychiatric symptoms. Such an association could potentially guide prediction, prevention and early intervention for children who are at risk of developing mental illness later in life.

## 1. Introduction

One in seven youth worldwide has a diagnosable mental health condition; however, most of this young population has not had appropriate interventions at a sufficiently early age to prevent further progression (World Health Organization, 2021). A family history of mental illness is linked to heightened risk of developing mental illness in youth, which may hinder their academic success, social interactions, and developmental progress (Garcia-Carrion et al., 2019). Thus, predicting at-risk children’s mental health is crucial for early intervention and improvement of youths’ life satisfaction.

In mental illness, cognitive dysfunction or impairment (e.g., problems with attention or memory relative to the general population) is a key predictor of symptoms and functioning (Green et al., 2019; Haining et al., 2022), including in youth (Morales-Muñoz et al., 2021). This highlights cognition as an early vulnerability marker and target for early interventions in at-risk youth. Cognitive dysfunction is often present before clinical symptoms emerge (Mollon et al., 2018) and is a core feature of many disorders (Abramovitch et al., 2021). Some differences in breadth, severity, and even direction of cognitive dysfunction have been noted within and across diagnostic categories, including (1) a general tendency toward reduced cognitive performance that varies by disorder (with schizophrenia typically showing the greatest impairment) and across individuals (with some patients being indistinguishable from controls regardless of diagnosis) (Abramovitch et al., 2021; Millan et al., 2012); (2) heightened attention to clinically meaningful stimuli in internalizing disorders, such as anxiety and depression (Lavigne et al., 2024); and (3) more targeted impairments in domains like attention and executive function in externalizing disorders, such as attention-deficit-hyperactivity disorder (Blanken et al., 2017). Nonetheless, individuals with mental illness, on average, perform worse in several cognitive domains relative to controls (Abramovitch et al., 2021), and to reflect this, current clinical guidelines recommend cognitive assessments as part of functional recovery planning for various psychological disorders (Catalan et al., 2021; East-Richard et al., 2020; Fett et al., 2022; Green et al., 2020; Malhi et al., 2021).

Cognitive impairments being prevalent and strong predictors of clinical and functional outcomes in many psychiatric disorders is coherent with transdiagnostic approaches to mental health. The Hierarchical Taxonomy of Psychopathology, which classifies psychiatric issues based on symptom components and similar psychological processes across disorders (Kotov et al., 2021), may better reflect the complexity and comorbidity of psychiatric problems than disorder-specific symptom profiles (McTeague et al., 2016). Studies in transdiagnostic clinical samples using clustering techniques have shown that cognitive impairment is a transdiagnostic phenomenon (Wenzel et al., 2024). Multiple systematic reviews and meta-analyses have also suggested that cognitive dysfunction is transdiagnostically evident, as noted above (Abramovitch et al., 2021; Cotter et al., 2018; Lavigne et al., 2024; Millan et al., 2012). The transdiagnostic nature of cognitive dysfunction is also apparent in the early stages of psychiatric disorders (Catalan et al., 2021; East--Richard et al., 2020; Fett et al., 2022; Wenzel et al., 2024). Thus, viewing cognitive impairments as key transdiagnostic targets may facilitate early clinical interventions, preserve cognitive skills and prevent cognitive decline. Moreover, relating transdiagnostic psychiatric symptoms to cognition may be more impactful than traditional disorder-specific classifications, enhancing the chances of functional recovery (Pantelis et al., 2015).

Psychiatric disorders often cluster in families, influenced by both genetic and environmental factors (Burmeister et al., 2008; Ortega Garcia et al., 2020). Youth with first-degree parental histories of psychiatric illness may face heightened risks due to both inherited vulnerabilities and exposure to adverse parental environments (Ramchandani and Stein, 2003). Previous meta-analyses reported impaired cognition in first-degree relatives of people with psychiatric disorders compared to those without (Calafiore et al., 2018; MacKenzie et al., 2019), further showing cognitive impairment as a key feature in the prediction of at-risk youth’s mental health progression. As cognitive impairment is a common and often prodromal feature of heritable psychiatric disorders, youth with first-degree relatives severely affected by mental illness (e.g., requiring hospitalization) are at higher risk for psychiatric disorders and may show early psychiatric symptoms. Given the prognostic value of cognition on psychiatric symptoms, identification of early cognitive differences in at-risk youth and associations with symptoms is essential for mitigating the onset and severity of future psychiatric disorders. Our study aimed to investigate the association between cognitive functioning and early psychiatric symptoms from a transdiagnostic perspective to better inform early identification and intervention strategies in at-risk youth.

We examined youth from the Adolescent Brain Cognitive Development (ABCD) study^®^ who were at risk for mental illness. Using multivariate partial least squares (PLS), we examined patterns of associations between cognitive performance (National Institutes of Health (NIH) Toolbox) (Weintraub et al., 2013) and transdiagnostic symptomatology in individuals with a first-degree relative previously hospitalized for mental illness. This multivariate approach allowed us to identify patterns capturing subtle associations between symptoms and cognition in at-risk youth, with the aim of informing early intervention strategies that may disrupt trajectories leading to more severe clinical progressions. Symptoms were categorized into psychosis, externalizing, and emotional dysfunction (internalizing) dimensions following HiTOP (Kotov et al., 2021), and cognitive measures into specific domains (e.g., attention, working memory, episodic memory, executive function, processing speed, expressive and receptive language) as defined within the NIH Toolbox. Such domains have been employed in recent literature, for example, suggesting that poorer executive function is associated with current and future internalizing symptoms in youth and early adolescence (Karr et al., 2024; Vedechkina et al., 2023), providing a sound basis for our investigation. This research aimed to (1) compare the performance of at-risk youth and controls on cognitive domains and HiTOP dimensions; (2) identify patterns between cognitive functioning and early psychiatric symptoms in at-risk youth. We hypothesized that (1) at-risk youth would perform worse cognitively and exhibit more early psychiatric symptoms compared to controls; and (2) PLS would identify at least one pattern linking early symptoms and cognitive functioning among at-risk children. Identifying such patterns before frank cognitive dysfunction or symptoms emerge would enhance our understanding of how subtle cognitive changes may drive psychiatric symptoms in youth and inform early intervention.

## 2. Methods

### 2.1. Participants

Our data were obtained from the ABCD Study^®^ (Michelini et al., 2019) 5.0 data release (June 2023, https://nda.nih.gov/study.html?id=2147), the largest long-term study of brain development and child health in the United States, including approximately 11,800 children recruited at age nine to ten. This study was not pre-registered. The NDA study (#2845) is available online: https://dx.doi.org/10.15154/z563-zd24.

We were interested in investigating cognitive performance and early psychiatric symptoms in at-risk youth with first-degree relative(s) previously hospitalized due to mental illness. The first round of filtering excluded participants without a direct family history of mental illness and retained 1109 participants with hospitalized first-degree relative(s) as our initial at-risk group. The second round of filtering excluded participants with missing data on our measures of interest (cognition, psychiatric symptoms, sociodemographics). After both rounds of filtering, 924 at-risk youth aged nine to ten remained.

Using the “MatchIt” package in R (Ho et al., 2021), we matched the at-risk group with 924 youth who had no first-degree relatives ever hospitalized due to mental illness on age, sex, and socioeconomic status to serve as controls. In the end, we selected a total of 1848 participants, including 924 at-risk (484 males and 440 females) with a mean age of 9.46 (SD = 0.51) and 924 controls (480 males, 444 females) with a mean age of 9.45 (SD = 0.51).

### 2.2. Measures

Cognitive performance was measured using the NIH Toolbox Cognition Battery, a multidimensional set of brief measures assessing cognitive function, validated for children and adolescents (Gershon et al., 2010; Varma et al., 2013). The total in-person administration time was approximately 35 min (Thompson et al., 2019). The NIH Toolbox yields individual scores for seven tasks measuring specific cognitive domains. (1) “Dimensional Change Card Sort” assesses executive function (e.g. cognitive flexibility) and attention and involves matching images by shape or color, with the matching dimension changing regularly (Zelazo et al., 2013). (2) “Flanker Inhibitory Control and Attention” measures executive control (e.g. inhibitory control) and attention, requiring participants to identify the direction of a central arrow flanked by congruent or incongruent arrows (Zelazo et al., 2013). (3) “Picture Sequence Memory” measures episodic memory and involves reproducing an arbitrarily ordered sequence of images presented on a computer (Bauer et al., 2013). (4) “List Sorting Working Memory” indexes working memory and requires participants to sort and sequence visual and auditory stimuli (Tulsky et al., 2014). (5) “Pattern Comparison” assesses processing speed by asking participants to identify whether two visual patterns are the same or not (Carlozzi et al., 2014). (6) “Picture Vocabulary” measures receptive language (e.g. vocabulary skills) and requires participants to identify the meaning of a word by matching a word they hear to one of four pictures on screen (Gershon et al., 2014). Finally, (7) “Oral Reading Recognition” indexes expressive language (e.g. reading decoding skills) and involves pronouncing or identifying a letter or word on screen (Gershon et al., 2013). We used the age-corrected standard scores and baseline data for all measures.

Psychiatric symptoms were measured using the Kiddie Schedule for Affective Disorders and Schizophrenia – Computerized Version (K-SADS-COMP; Townsend et al., 2020). The K-SADS-COMP consists of 25 self-administered modules, each assessing symptoms related to a psychopathological syndrome (e.g., depression, mania). At baseline, caregivers completed all modules except enuresis and encopresis, tic disorders, and selective mutism, whereas children completed the following modules only (supported by trained staff): depression, mania, disruptive mood regulation disorder, social anxiety disorder, generalized anxiety disorder, sleep problems, and suicidality (Barch et al., 2021). In the ABCD baseline data, only presence (1) and absence (0) of symptoms were recorded, and these were used to calculate total syndrome scores. In cases where both child and caregiver scores were available (e.g., depression), we scored a symptom as present if either the child or their caregiver reported presence. As such, the maximum possible syndrome score was equivalent to the total number of symptoms for that syndrome.

In line with the well-established HiTOP model (Kotov et al., 2021), which emphasizes the dimensional organization of psychopathology, we summed the K-SADS-derived syndrome scores into the three HiTOP superspectra: (1) “Emotional Dysfunction”, consisting of syndrome scores (sums of presence/absence of underlying symptoms) from the following K-SADS syndromes: depression, panic disorder, agoraphobia, separation anxiety, social anxiety disorder, specific phobia, generalized anxiety disorder, obsessive-compulsive disorder, eating disorders, post-traumatic stress disorder, sleep problems, and suicidality; (2) “Psychosis,” including mania and psychosis; (3) “Externalizing,” including disruptive mood dysregulation disorder, attention deficit hyperactivity disorder, oppositional defiant disorder, conduct disorder, autism spectrum disorder, alcohol use disorder, and substance use disorders. Due to the differences in the number of symptoms assessed per syndrome – and the number of syndromes per HiTOP dimension – the range of maximum possible scores naturally varied across HiTOP dimensions (Emotional dysfunction: 0–248; Psychosis: 0–78; Externalizing: 0–448). This was addressed during analysis as described below.

### 2.3. Statistical analysis

We acquired the ABCD Study^®^ 5.0 data release through the NIMH Data Archive. We merged each participant’s baseline data according to their subject IDs (including demographics, scores for NIH Toolbox tasks and HiTOP spectra) using R (version 4.5.1) (RCoreTeam, 2021), RStudio (Version, 2025.05.1, Build 513), and in-house scripts.

#### 2.3.1. Data analysis

After using the Shapiro Wilk normality test, we found that the NIH Toolbox and HiTOP values were not normally distributed for either group (see Supplementary Figs. S1–S4). Thus, statistical comparisons between the mean performance of each cognitive domain and HiTOP dimension were performed using the Mann Whitney *U* Test (Wilcoxon Rank Sum Test, α = 0.05). As these groups comparisons were completed within each HiTOP dimension separately, the notable range differences did not impact these analyses. Means and standard deviations were generated using the “Table 1” package (Rich, 2023) in R Studio. Spearman’s rank correlations (α = 0.05) were performed to obtain the correlation coefficients between cognition (cognitive domains) and psychiatric symptoms (HiTOP dimensions) for the at-risk and control groups separately.

We then performed partial least squares (PLS) to further explore the correlational patterns between cognitive domains and HiTOP dimensions in the at-risk group only. PLS involves computing a correlation matrix between sets of variables (i.e., 7 cognitive domains and 3 HiTOP dimensions) and performing singular value decomposition (principal component analysis) on the resulting matrix to identify patterns of associations (Abdi and Williams, 2013). As a first step, PLS standardizes all variables so that each has a mean of 0 and a standard deviation of 1, ensuring that comparisons between measures with different scales (i.e., HiTOP dimensions) are sound. Components (or latent variables) were then extracted from the pattern of correlations between the 7 cognitive and 3 HiTOP spectra scores and interpreted based on their respective loadings.

**Fig. 1.**
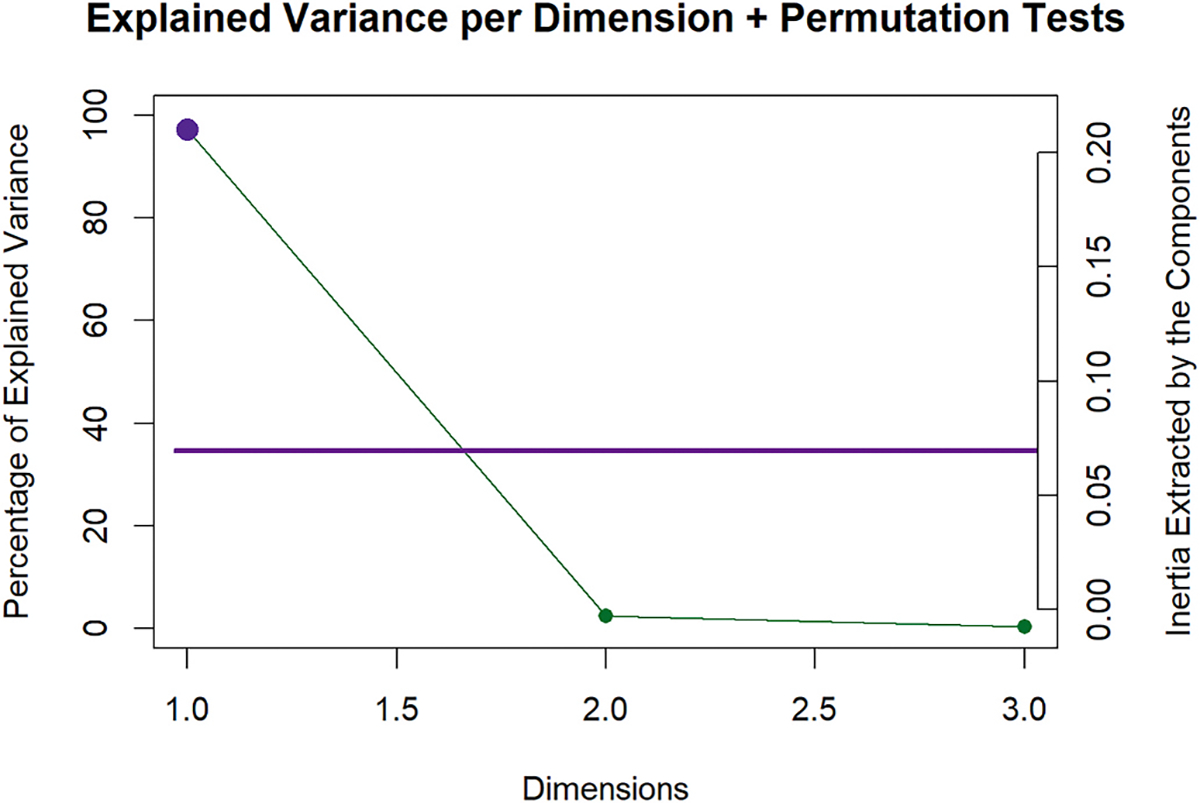
Scree plot of explained variance per component and significance (permutation test).

A permutation test (n = 1000) was performed to assess the significance of the PLS latent variables (α = 0.05), and bootstrap estimations of standard errors (n = 1000) assessed the reliability of each input variable using a 95% confidence interval. Using the R packages data4PCCAR (Beaton and Abdi, 2024) and PTCA4CATA (Abdi, 2024), we performed the permutation test and generated a scree plot (Fig. 1), which demonstrates the explained variance per component, with the significance line cut-off illustrating the number of significant components. Using the R package TExPosition (Beaton et al., 2014), we generated figures showing the bootstrap ratios for the HiTOP dimensions and NIH Toolbox cognitive domains, indicating measures within the two sets that significantly contributed to the identified pattern(s); these are presented by the bars that exceed the dotted significance cut-off line. An alpha level of 0.05 was used for all statistical tests.

## 3. Results

Means and standard deviations for cognitive measures and transdiagnostic HiTOP dimensions are displayed in Table 1 for at-risk youth and controls separately. Group comparisons of cognitive and clinical scores within each test and domain are also displayed in Table 1, showing at-risk youth with significantly lower scores than controls on the Total Composite Score, Dimensional Change Card Sort, and Picture Sequence Memory as well as significantly greater subsyndromal symptom severity in all HiTOP dimensions.

Among at-risk youth, there were significant negative correlations between: (1) Emotional Dysfunction and all cognitive domains except Pattern Comparison and Flanker, (2) Psychosis and all cognitive domains except Flanker; and (3) Externalizing and all cognitive domains except Pattern Comparison. Among control youth, significant negative correlations were observed between: (1) Emotional Dysfunction and Dimensional Change Card Sort and Oral Reading Recognition; (2) Psychosis and Picture Vocabulary and Oral Reading Recognition; and (3) Externalizing and Dimensional Change Card Sort, Picture Sequence Memory, Pattern Comparison, Picture Vocabulary, and Oral Reading Recognition (see Tables 2 and 3).

**Fig. 2.**
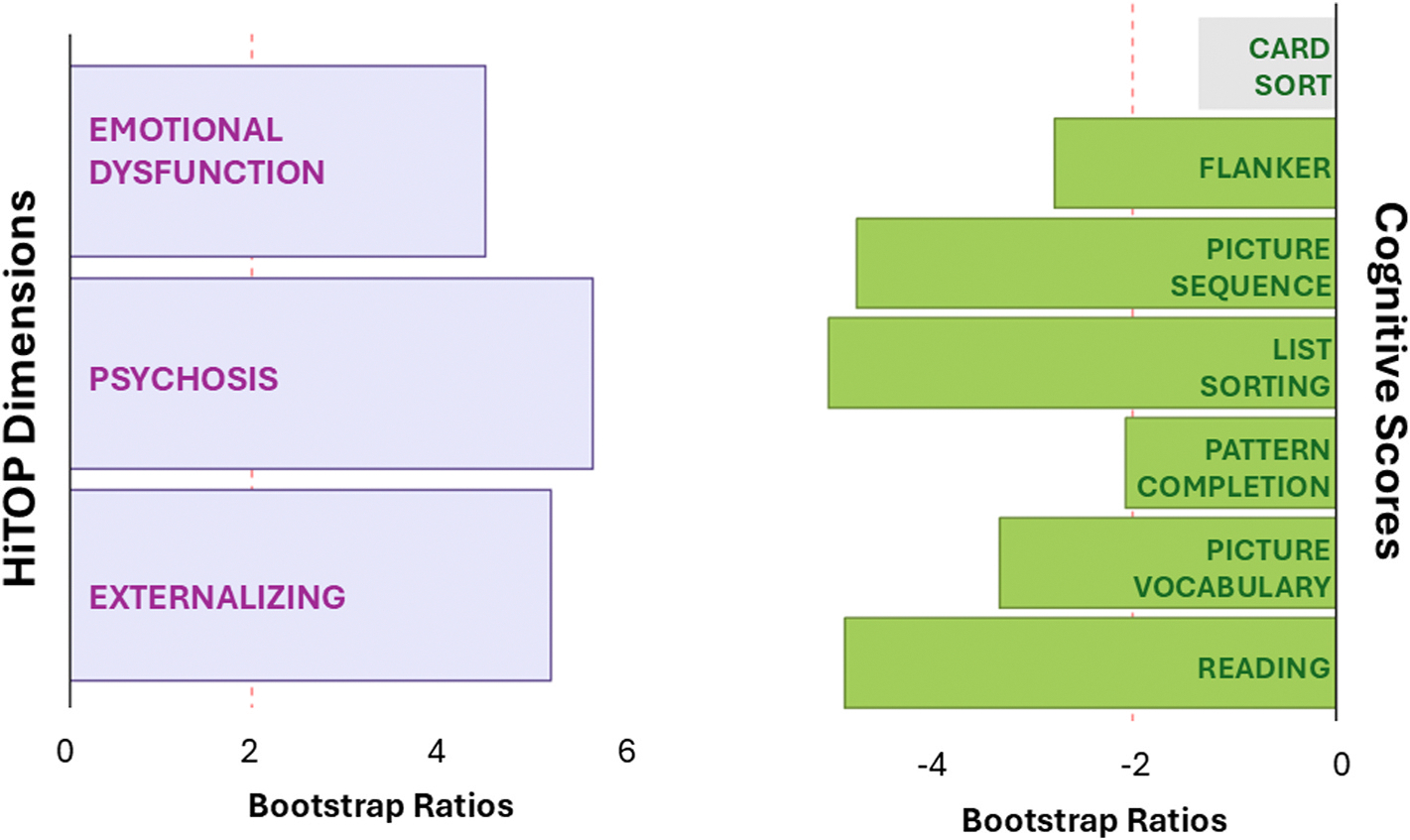
Bootstrap ratios for each symptom dimension and cognitive measure.

PLS analysis was used to identify latent patterns of associations between early psychiatric symptoms (HiTOP dimensions) and cognitive functioning (NIH Toolbox cognitive domains) in at-risk youth. Among the three possible latent variables (components), one was significant, accounting for approximately 97.38 % of the shared variance between the two sets of variables (see Fig. 1). The non-significant latent variables accounted for 2.32 % and 0.30 % of the shared variance. All HiTOP dimensions showed positive loadings and reached significance (bootstrap ratio cut-off ±1.96) on the first component: psychosis (5.62), externalizing (5.17), and emotional dysfunction (4.47). All cognitive domains showed negative loadings: List Sorting (−4.89), Oral Reading Recognition (−4.73), Picture Sequence Memory (−4.62), Picture Vocabulary (−3.24), Flanker Inhibition Control (−2.71), Pattern Comparison (−2.02), and Dimensional Card Sort (−1.32), of which all but Dimensional Change Card Sort reached significance (see Fig. 2). We, thus, identified one dominant pattern linking cognitive performance and transdiagnostic symptom dimensions in at-risk youth, driven cognitively by episodic memory, working memory, and both receptive and expressive language.

## 4. Discussion

This study aimed to explore patterns of associations between cognitive performance and early psychiatric symptoms in youth at high familial risk of severe mental illness. Our findings suggest that (1) at-risk youth present significantly lower cognitive scores compared to controls in executive function and episodic memory and exhibit significantly higher, albeit subsyndromal, psychiatric symptoms than controls across all HiTOP dimensions; (2) in at-risk youth especially, poorer cognitive performance is associated with more psychiatric symptoms; and (3) multivariate associations between cognition and symptoms in at-risk youth are best understood by a general pattern linking early psychiatric symptoms and poorer cognitive performance, driven by episodic memory, working memory, and language abilities. Despite few significant cognitive differences between at-risk children and controls, our multivariate analysis captured the presence of subtle, early cognitive differences in at-risk youth – potentially prodromal in nature – which are already significantly associated with transdiagnostic symptoms across all three HiTOP dimensions. These findings suggest that subtle cognitive changes may be present early on and linked to psychiatric symptoms even prior to the emergence of clinically significant impairments, highlighting the importance of early intervention for prediction and prevention of mental illness, particularly in the language and memory domains.

Our comparisons revealed that at-risk youth exhibited significantly lower performance in executive function and episodic memory, supporting our hypothesis in these domains. Consistent with previous research, our findings highlight the critical influence of familial factors on cognitive development across the first two decades of life, particularly in domains such as executive function, memory, and complex reasoning. Such cognitive vulnerability is often accompanied by increased heritability of externalizing behaviors, anxiety, and depressive symptoms (Bergen et al., 2007; Haworth et al., 2010). Indeed, at-risk youth displayed significantly more psychiatric symptoms across all HiTOP dimensions in the current study—emotional dysfunction, psychosis, and externalizing symptoms—further corroborating the notion that they are more likely to exhibit elevated psychiatric symptoms compared to their peers without first-degree relative(s) who had previously been hospitalized due to psychiatric concerns. Our findings align with literature highlighting potential genetic and familial underpinnings of psychiatric disorders (Uher et al., 2023), which suggests that youth with first-degree relative(s) with mental illness are at increased risk for transdiagnostic psychiatric disorders, reinforcing the critical need for early identification and targeted intervention in this vulnerable youth population.

Despite the limited cognitive differences observed between at-risk youth and controls, correlations revealed significant negative associations between cognitive performance and the different HiTOP dimensions, particularly in at-risk youth. Moreover, our multivariate analysis identified a dominant pattern between all HiTOP dimensions and cognitive domains that accounted for 97 % of the variance in associations between psychiatric symptoms and cognitive functioning within the at-risk youth sample. Cognitively, this pattern was primarily driven by lower episodic memory (Picture Sequence Memory), working memory (List Sorting Working Memory), and both receptive (Picture Vocabulary) and expressive (Oral Reading Recognition) language abilities. Prior literature has tended to focus on specific psychiatric symptoms or disorders when investigating links with cognition in at-risk youth. A previous review found poorer episodic memory in youth at risk for psychosis and identified episodic and working memory as key predictors of conversion to psychosis (Seabury and Cannon, 2020). Another review observed a transdiagnostic pattern of lower executive function (including working memory) in externalizing disorders (Sadozai et al., 2024). Regarding language abilities, studies have primarily linked early language difficulties to psychosis risk and internalizing behaviors (Bornstein et al., 2013; Girard et al., 2022), though some work has implicated externalizing symptoms as well (Hentges et al., 2021). Our findings suggest that cognition, especially memory and language-related difficulties, may serve as a broader developmental predictor than previously recognized, particularly during early youth and prior to the onset of explicit symptoms and/or frank cognitive impairments within at-risk youth. This offers valuable insights for early identification and intervention strategies targeting emerging psychiatric symptoms among youth who are more susceptible to developing psychiatric symptoms due to family history. Taken together with the limited group differences identified, particularly within the language domains, these findings suggest that even small and possibly undetectable differences can impact subtle psychiatric symptoms in at-risk youth.

Strengths of this study include a large sample of 924 at-risk youth and 924 matched controls, a well-characterized methodology, and a transdiagnostic framework applicable to a broad youth population with diverse psychiatric symptoms. While this generalizability enhances the study’s applicability, it may come at the expense of specificity for individual diagnoses. However, our approach aligns with growing efforts towards transdiagnostic clinical staging models, which highlight the importance of early transdiagnostic symptoms in youth that may progress into more clearly defined psychiatric disorders in emerging adulthood (McGorry et al., 2024).

A limitation of this work is that only symptom presence or absence was recorded and, as such, our findings refer specifically to the number of symptoms present, rather than their severity. In addition, our findings likely rest on potentially subtle and small effects, due to the at-risk sample, their young age, and the heterogeneity of parental disorder diagnoses. However, these limitations were necessary given our aim to identify patterns of associations between cognition and early psychiatric symptoms, to drive future work in early detection and intervention of mental illness. Given the young age of our sample, it remains uncertain whether the subtle cognitive difficulties in sampled at-risk children represent an early sign of future impairment or a delay in cognitive development. Future studies should consider replicating this analysis using clinical samples, samples spanning a wider age range, and/or conducting longitudinal analyses to track changes in cognitive performance and psychiatric symptoms (and their associations) over time. While this study provides a broad perspective on family history of mental illness as a risk factor for children, it cannot distinguish between the specific effects of nature and nurture during the upbringing of at-risk youth, such as parenting practices and attachment styles (Andreassen et al., 2023; Kerns and Brumariu, 2014). Therefore, future research should also focus on identifying detailed risk and protective factors to better understand and support youth at higher risk of developing mental illness.

Previous research has demonstrated that early intervention, when problematic signs are first identified, can reduce the prevalence and severity of adverse outcomes while contributing to the efficient and effective use of health resources (McGorry and Mei, 2018). Earlier studies have also highlighted that familial influences on cognitive performance increase with age (Haworth et al., 2010), urging the need for early intervention at a young age. Building on these findings, we hope our study can serve as a foundational step for future longitudinal research using the ABCD sample and others to investigate the trajectory and predictive patterns of transdiagnostic psychiatric symptoms. Ultimately, we hope our study can inform future research on youth mental health, advocate for early cognitive and psychiatric interventions for at-risk children, and support initiatives to promote timely cognitive development in children at heightened risk for psychological disorders.

## Supplementary Material

1

## Figures and Tables

**Table 1:** Demographics and comparisons between cognitive functioning and psychiatric symptoms in youth.

	Groups				Comparison	
	At Risk (924)		Control (924)			
				
	M	SD	M	SD	U value	P value

**Cognitive Domains**						
Total composite score	97.2	16.9	99.1	18.7	402027	0.030*
Fluid composite	92.9	17.0	94.6	18.0	404437	0.050
Dimensional Change Card Sort	94.6	14.5	95.7	14.4	404095	0.045*
Flanker Inhibitory Control	94.8	13.9	95.4	13.8	415051	0.299
Picture Sequence Memory	99.0	15.3	101.0	16.1	401737	0.028*
List Sorting Working Memory	98.5	13.8	100.0	15.5	404793	0.053
Pattern Comparison	92.3	22.8	92.6	22.3	424624	0.843
Crystallized composite	103.0	17.1	104.0	18.6	406405	0.073
Picture Vocabulary	105.0	16.2	106.0	18.3	406736	0.070
Oral Reading Recognition	100.0	17.8	101.0	18.1	410820	0.159
**HiTOP dimensions**						
Emotional	22.2	21.7	14.5	16.2	530026	<0.001***
Dysfunction						
Psychosis	5.6	6.8	3.8	5.6	495970	<0.001***
Externalizing	18.9	20.2	12.2	16.4	517134	<0.001***

**Table 2:** Correlations between cognitive performance and psychiatric symptoms in at-risk youth.

NIH Toolbox Measures	HiTOP Dimensions		
	Emotional Dysfunction	Psychosis	Externalizing

Dimensional Change Card Sort	−0.07*	−0.07*	−0.10**
Flanker Inhibitory Control and Attention	−0.04	−0.04	−0.09**
Picture Sequence Memory	−0.10**	−0.12***	−0.14***
List Sorting Working Memory	−0.11**	−0.12**	−0.16**
Pattern Comparison	−0.04	−0.08*	−0.04
Picture Vocabulary	−0.14***	−0.12***	−0.11**
Oral Reading Recognition	−0.12***	−0.12**	−0.15**

*Note:* Statistical significance is marked by * (p < 0.05), ** (p < 0.01) *** (p < 0.001).

**Table 3:** Correlations between cognitive performance and psychiatric symptoms in control youth.

NIH Toolbox Measures	HiTOP Dimensions		
	Emotional Dysfunction	Psychosis	Externalizing

Dimensional Change Card Sort	−0.12***	−0.06	−0.15***
Flanker Inhibitory Control and Attention	−0.03	−0.02	−0.05
Picture Sequence Memory	−0.05	−0.06	−0.11**
List Sorting Working Memory	−0.05	−0.06	−0.04
Pattern Comparison	−0.05	−0.04	−0.09**
Picture Vocabulary	−0.06	−0.07*	−0.08*
Oral Reading Recognition	−0.09**	−0.07*	−0.12**

*Note:* Statistical significance is marked by * (p < 0.05), ** (p < 0.01) *** (p < 0.001).

## Appendix A. Supplementary data

Supplementary data to this article can be found online at https://doi.org/10.1016/j.jpsychires.2025.12.017.
