# Supplementary material for: Back to the future: Linking early psychiatric symptoms to transdiagnostic cognitive functioning in at-risk youth from the adolescent brain cognitive development study
Source: J Psychiatr Res. Author manuscript; Available in PMC 2026 Jul 24. (PMC13399204; doi:10.1016/j.jpsychires.2025.12.017)
Supplement: 1 [file NIHMS2188335-supplement-1.docx]

**Back to The Future: Linking Early Psychiatric Symptoms to Transdiagnostic Cognitive Functioning in At-Risk Youth from the Adolescent Brain Cognitive Development Study**

Supplementary Material

Wang, C.J.^1,2^, Raucher-Chéné, D.^2,3±^, & Lavigne, K.M.^2,3±*^

^1^ Department of Psychology, McGill University, Montreal, Quebec, Canada

^2^ Douglas Research Centre, Montreal, Quebec, Canada

^3^ Department of Psychiatry, McGill University, Montreal, Quebec, Canada

± Delphine Raucher-Chéné and Katie Lavigne share senior authorship.

* Corresponding Author: Katie M. Lavigne. F-1101.2, 6875 Boulevard LaSalle, Frank B. Common Pavilion, Douglas Research Centre, Montreal, QC, H4H 1R3, Canada. E-mail: [katie.lavigne@mcgill.ca](mailto:katie.lavigne@mcgill.ca), Phone: 1-514-761-6131 x 6251.

Figure S1. Histograms, skewness, and kurtosis for cognitive performance in at-risk youth.


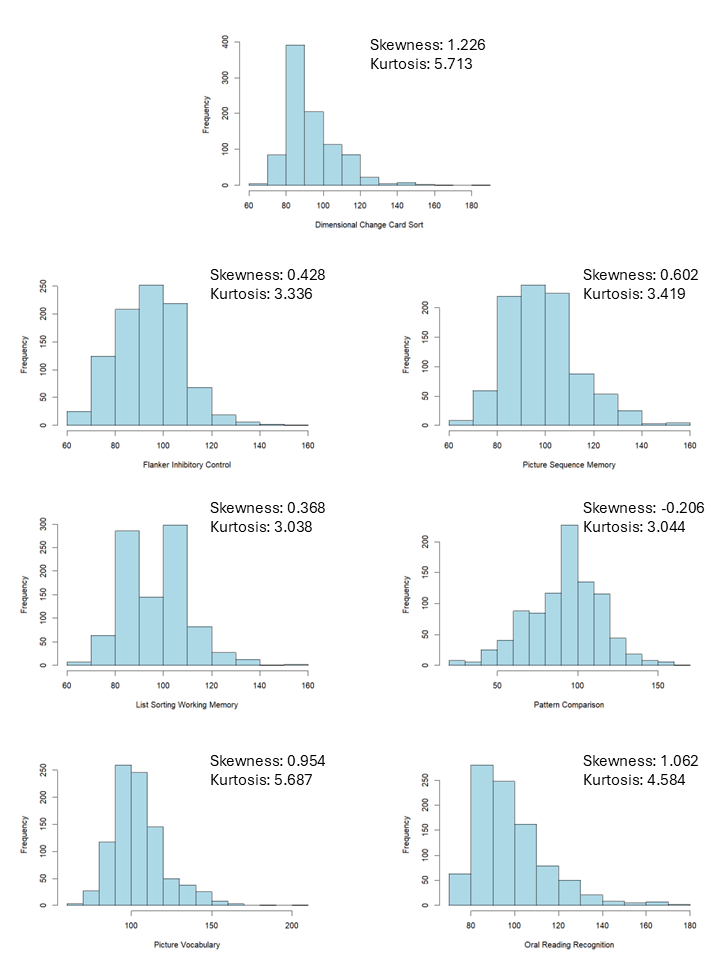


Figure S2. Histograms, skewness, and kurtosis for psychiatric symptoms in at-risk youth.


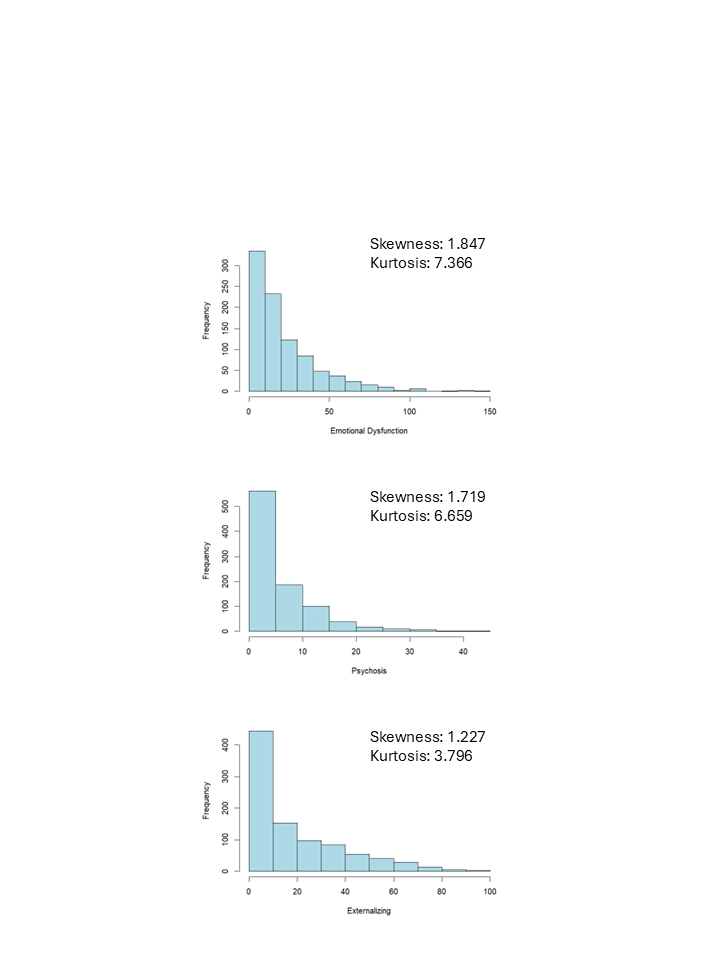


Figure S3. Histograms, skewness, and kurtosis for cognitive performance in control youth.


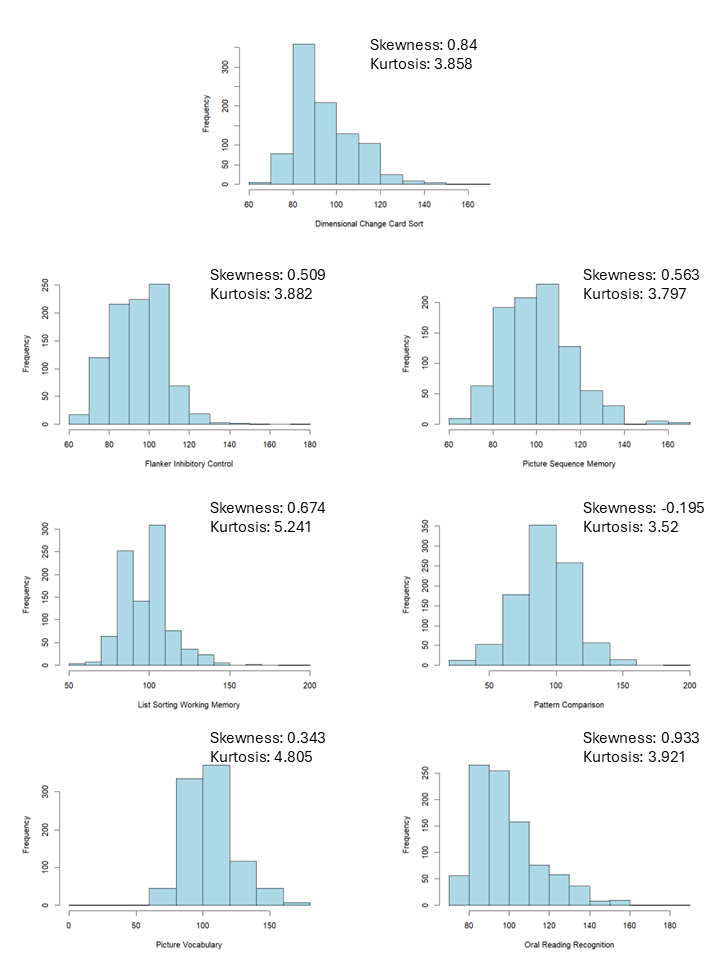


Figure S4. Histograms, skewness, and kurtosis for psychiatric symptoms in control youth.

**
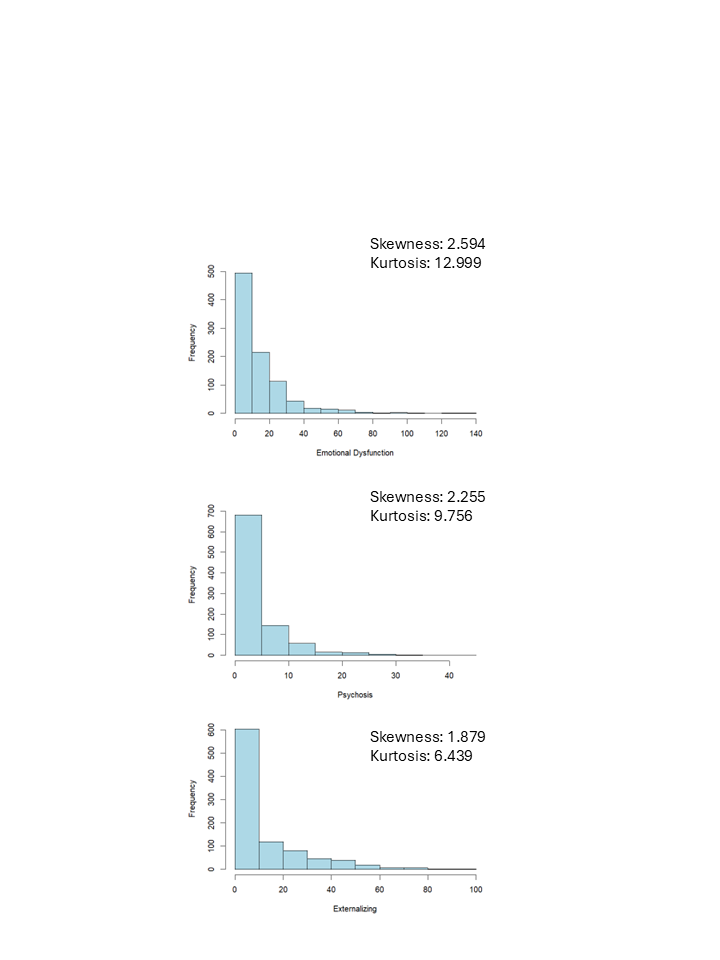
**
